# Stem cell-like circulating tumor cells identified by Pep@MNP and their clinical significance in pancreatic cancer metastasis

**DOI:** 10.3389/fonc.2024.1327280

**Published:** 2024-06-25

**Authors:** Xiangyu Chu, Xiejian Zhong, Shouge Zang, Mengting Wang, Ping Li, Yongsu Ma, Xiaodong Tian, Yanlian Yang, Chen Wang, Yinmo Yang

**Affiliations:** ^1^ Department of Hepatobiliary and Pancreatic Surgery, Peking University First Hospital, Beijing, China; ^2^ Chinese Academy of Sciences Key Laboratory of Biological Effects of Nanomaterials and Nanosafety, Chinese Academy of Sciences Key Laboratory of Standardization and Measurement for Nanotechnology, Chinese Academy of Sciences Center for Excellence in Nanoscience, National Center for Nanoscience and Technology, Beijing, China; ^3^ Department of General Surgery, Fuyang People's Hospital of Anhui Medical University, Fuyang, China; ^4^ School of Nanoscience and Engineering, University of Chinese Academy of Sciences, Beijing, China

**Keywords:** pancreatic cancer, peptide-functionalized nanomagnetic beads, circulating tumor cells, CXCR4 over-expression, early recurrence, liver metastasis

## Abstract

**Objective:**

The circulating tumor cells (CTCs) could be captured by the peptide functionalized magnetic nanoparticles (Pep@MNP) detection system in pancreatic ductal adenocarcinoma (PDAC). CTCs and the CXCR4 expression were detected to explore their clinical significance. The CXCR4+ CTCs, this is highly metastatic-prone stem cell-like subsets of CTCs (HM-CTCs), were found to be associated with the early recurrence and metastasis of PDAC.

**Methods:**

CTCs were captured by Pep@MNP. CTCs were identified via immunofluorescence with CD45, cytokeratin antibodies, and the CXCR4 positive CTCs were assigned to be HM-CTCs.

**Results:**

The over-expression of CXCR4 could promote the migration of pancreatic cancer cell *in vitro* and *in vivo*. In peripheral blood (PB), CTCs were detected positive in 79.0% of all patients (49/62, 9 (0–71)/2mL), among which 63.3% patients (31/49, 3 (0–23)/2mL) were HM-CTCs positive. In portal vein blood (PVB), CTCs were positive in 77.5% of patients (31/40, 10 (0–40)/2mL), and 67.7% of which (21/31, 4 (0–15)/2mL) were HM-CTCs positive CTCs enumeration could be used as diagnostic biomarker of pancreatic cancer (AUC = 0.862), and the combination of CTCs positive and CA19–9 increase shows improved diagnostic accuracy (AUC = 0.963). in addition, PVB HM-CTCs were more accurate to predict the early recurrence and liver metastasis than PB HM-CTCs (AUC 0.825 vs. 0.787 and 0.827 vs. 0.809, respectively).

**Conclusions:**

The CTCs identified by Pep@MNP detection system could be used as diagnostic and prognostic biomarkers of PDAC patients. We identified and defined the CXCR4 over-expressed CTC subpopulation as highly metastatic-prone CTCs, which was proved to identify patients who were prone to suffering from early recurrence and metastasis.

## Introduction

Pancreatic ductal adenocarcinoma (PDAC) is one of the most malignant and lethal digestive system neoplasm with 5-year survival rate lower than 10% (1). In China, approximately 88,000 patients died of PDAC in 2016 (2). The poor prognosis was attributed to the insidious onset and late diagnosis. At the time of diagnosis, approximately half of the patients have distant metastases (3). And 80% of patients who undergo radical surgery experience recurrence at remote or local sites within 2 years (4). Current radiological examination and blood tests (carbohydrate antigen 19–9) could not identify pancreatic cancer patients who may suffer postoperative recurrence and metastasis. Therefore, it is necessary to develop precise diagnostic and prognostic biomarkers to identify the patients who are prone to suffering postoperative early recurrence and metastasis due to the limited sensitivity and specificity of the current examinations. Circulating tumor cells (CTCs) are tumor cells shed from the primary tumor or metastatic foci in the blood, containing the real tumor biological information (5, 6). The CTCs can fall from the tumor into the circulatory system at the early stage, and it could form tiny metastatic foci in the “soil” of pre-metastatic niche formation (7). The highly malignant biological behavior of CTCs might lead to early occult metastasis in some early-stage patients and thus resulting in postoperative recurrence and metastasis. Therefore, the capture and detection of CTCs and CTC subsets could be used to diagnose, predict the onset of distant metastasis and evaluate the prognosis of epithelial malignancies such as breast, colorectal, and pancreatic cancers (8–10).

The CellSearch® detection system is designed to capture CTCs in whole blood (7.5 mL) based on a 10-nm ferromagnetic fluid modified by epithelial cell adhesion molecule (EpCAM) antibody, which is the FDA-approved CTC detection method (11). However, the CTCs detection rate in the peripheral blood (PB) of pancreatic cancer patients is only 7–48% at different stages (12). Our team previously developed the novel nanomagnetic bead assay (Pep@MNPs), which could capture the CTCs by using EpCAM-targeting peptide functionalized magnetic nanoparticles. We *de novo* designed the EpCAM-targeted peptide, Pep10, whose binding affinity to EpCAM (1.98 ×10^−9^ mol L^−1^) was similar to anti-EpCAM antibody (2.69×10^−10^ mol L^−1^). The CTC isolation method (Pep@MNPs) was designed by using the EpCAM-targeted peptide functionalized iron oxide magnetic nanoparticles (13). This technique could detect E-CTCs based on EpCAM-targeted peptide The capture efficiency and purity were higher than those of CellSearch® due to the higher density and well orientation of target peptides modified on the surface of magnetic nanoparticles (13–15). It could effectively detect CTCs in only 2 mL of whole blood, whose capture efficiency and purity was proved to reach to 90% and 93% in breast, prostate, and liver cancers (13). Lower synthesis cost of peptides in Pep@MNPs assay was another attractive advantage as compared to CellSearch® system in clinical applications.

It was reported that CTC counts are important for the prognosis evaluation and treatment strategy selection of cancer patients, and CTCs were enriched with important information about tumor biology (16–18). The CTCs could be classified into different subgroups based on biomarkers, thus interpreting their biological information which could help identify CTCs subtypes with highly malignant behavior. The transitional CTCs (vimentin^+^ CTCs) were identified and presumed to mediate cancer metastasis. The transitional CTCs were highly correlated with the late recurrence in pancreatic cancer patients (area under curve = 0.72). The transitional CTC positivity was associated with higher subsequent recurrence rates, different recurrence patterns and poorer recurrence-free survival (19, 20). CTC counts and vimentin+ CTCs were also significantly associated with response to chemotherapy in pancreatic cancer (21). The HER2-positive CTCs were associated with worse overall survival (OS) and recurrence-free survival (RFS) in breast cancer (22). And they could be used to identify a subset of breast cancer patients with high HER2 expression and dynamically evaluate the efficacy of anti-HER2 therapy during treatment (23–26). In summary, CTCs with different biomarkers could be used to identify patients with different malignancy tumor biology, thus help translate the interpretation of biological information into diagnosis and treatment evaluation.

The C-X-C motif chemokine receptor 4, CXCR4, is a chemokine stromal cell-derived factor 1α (CXCL12)-specific receptor, played an important role in promoting the PDAC progression. Studies reported that the expression of CXCR4 was strongly associated with local and distant recurrence, metastasis and poor prognosis in PDAC patients (27, 28). CXCR4 could promote the proliferation and invasion of PDAC by activating the AKT signaling pathway (29, 30). Singh et al. reported that CXCL12 can induce sonic hedge-hog (SHH) gene expression in pancreatic cancer cells through CXCR4 activation of the downstream Akt and ERK pathways (31). Saur et al. (32) revealed that CXCR4 expression significantly increases the metastatic potential of pancreatic cancer cells *in vivo*, leading to liver and lung metastases in mice. Large amounts of the chemokine CXCL12 are produced by organs normally affected by cancer metastasis, such as the lungs and liver (33). This can attract CXCR4-positive tumor cells to specific metastatic sites (33). Thus, it is possible that CXCR4-expressing tumor cells could be prone to forming metastatic tumors in the liver and lungs. In the present study, our data analysis implied that the over-expressed genes of PDAC patients were significantly enriched in the cell adhesion, cell-cell signaling, positive regulation of migration and positive regulation of proliferation signaling pathways. We focused on the CXCR4 gene, and existing studies suggested that CXCR4 could promote the progression and liver metastasis of PDAC, which was also verified in our research. Therefore, we defined the CXCR4 over-expressed CTC subpopulation as highly metastatic-prone CTCs (HM-CTCs), which could help identify patients who were prone to suffering early recurrence and metastasis before surgery.

CXCR4 may also influence the PDAC tumor immune microenvironment (TIME) and the CXCR4 inhibitor could improve the prognosis of PDAC by regulating the TIME. Bockorny et al. reported that the combined blockade of CXCR4 and PD-1 enhanced the chemotherapeutic effect in metastatic PDAC (NCT02826486) (34). Nanoparticles-based preclinical studies also implied that the combination of CXCR4 inhibitor and anti-PD1 could enhance the immune response by increasing the infiltration of CD4+ T cells, CD8+ T cells and polarization of macrophages, thus significantly inhibiting the progression of PDAC (35, 36). Therefore, it is necessary to early identify CXCR4-high expression patients, whose prognosis may benefit from the combination of CXCR4 and immunochemotherapy.

Herein, we verified the function of CXCR4 gene *in vitro* and *in vivo*, which could promote the pancreatic cancer liver metastasis. Then we used the Pep@MNP platform to capture and detect CTCs and HM-CTCs in the PB and PVB of PDAC patients. HM-CTCs could help evaluate the occurrence of metastasis, early recurrence and prognosis in pancreatic cancer patients. It can be used to identify pancreatic cancer patients who can benefit from the combination of CXCR4-targeted therapy and immunochemotherapy. This investigation showed well application prospects for precise treatment of PDAC.

## Methods

### Cell culture and transfection

Nine cell lines (human pancreatic cancer cell lines AsPC-1, PANC-1, MIA-PaCa-2, BxPC-3, T3M4 and Capan-1; normal human pancreatic ductal epithelial cells (HPNE) and 293T cells) were all purchased from American Type Culture Collection (ATCC) (Manassas). The cells were cultured in different basic media supplemented with 100 U/ml penicillin-streptomycin (PS) (Gibco) and 10% fetal bovine serum (FBS) (Gibco), including DMEM medium (Gibco) for PANC-1, MIA-PaCa-2, Capan-1, 293T and HPNE and 1640 medium (Gibco) for AsPC-1 and T3M4. The cells were maintained at 37°C in an atmosphere with 5% CO_2_ and 95% moisture. The lentiviral vector for CXCR4 over-expression (OE-CXCR4) was constructed by SyngenTech. Lipofec-tamine™ 3000 was used to produce the lentiviruses by the transfecting the 293T cells. AsPC-1 cells were transfected using the Lentiviruses and screened in puromycin.

### Flow cytometry analysis

The FCM was performed to evaluate the expression of CXCR4 in cells. The cells were blocked by the 5% bovine serum albumin (BSA) (Sigma-Aldrich) for 30 min at 37°C. Then, the blocked cells were incubated with Alexa Fluor® 647-conjugated antibodies for 60 min at 37°C, including anti-CXCR4 (Abcam, ab216548), and IgG isotype controls. The BD Accuri™ C6 flow cytometer (BD Biosciences) was applied to detect the expression of CXCR4.

### Migration assay

AsPC-1 cells and OE-CXCR4 AsPC-1 cells were suspended in starvation media (1×10^6^ cells mL^-1^). Then 100 µL of the cells were plated in the upper chamber of the transwell chamber (Millipore) with a pore size of 8 µm in a 24-well plate, while 600 µL medium supplemented with normal FBS were added into the lower chamber and served as a chemotactic agent. Cells invading into the lower chamber were fixed after incubation for 48 h, stained with crystal violet, and recorded under the upright micro-scope (five fields per chamber). The crystal violet was washed by the 33% ethylic acid and collected in the 96-well plate. Then the optical absorbance of each well at 570 nm was measured using a microplate reader.

### Animal liver metastatic model

The 5×10^5^ AsPC-1 cells or OE-CXCR4 AsPC-1 cells were planted into the spleen to construct the pancreatic cancer liver metastatic mice (3 mice/group). On day 30, the mice were sacrificed, and their livers were collected and photographed. The metastatic nodules were counted and recorded.

### Patients and blood sample collection

Sixty-two pancreatic cancer patients at the Peking University First Hospital between September 2019 and September 2021 and ten healthy subjects were enrolled in our study. The inclusion criteria for pancreatic cancer patients were (1) voluntary participation and signing of the informed consent form, (2) postoperative or puncture pathology confirmed as PDAC, and (3) the willing to observe the study’s follow-up protocol. The exclusion criteria included (1) a history of other malignant tumors and (2) a history of surgery, radiotherapy, chemotherapy, or immunotherapy for PDAC. Our multidisciplinary team developed treatment strategies for patients with pancreatic diseases. All the surgeries were performed by the same team.

Demographic data, laboratory tests, imaging tests, and pathology reports, including sex, age, carbohydrate antigen 19–9 (CA19–9), tumor staging, margin status, and lymph node status, were accurately recorded. During the follow-up period, CA 19–9 levels and abdominal pelvic enhancement on CT or MRI were routinely evaluated. In addition, chest CT, PET-CT, and other examinations were performed if necessary. CA19–9 was measured in the certified clinical laboratory in the Peking University First Hospital. This study was approved by the Ethics Committee of the Peking University First Hospital.

The participants’ fasting peripheral blood was collected (2 mL) in an EDTA-containing tube before surgery or systemic therapy. For patients who underwent radical surgery, the operator obtained 2 mL of PVB from a few centimeters above the splenic and superior mesenteric vein confluences after evaluating the hepatic portal anatomy (**Figure S1**). A 23-gauge syringe or butterfly needle, with both wings cut off, was used to puncture the portal vein during laparoscopic or open surgery. A previous study demonstrated no significant difference in portal CTC counts before and after resection (37). Nonetheless, excessive intraoperative tumor compression should be cautiously avoided. Compression to stop the bleeding was sufficient for the puncture site. All PBs and PVBs were assessed within 4 hours of incubation at room temperature.

### Detection and enumeration of CTCs

The use of Pep@MNPs to detect and enumerate CTCs was described in our previous studies (22, 23). Briefly, Pep@MNPs (10 μL) were added to 2 mL of PB or PVB and incubated at room temperature on a horizontal shaker at 160 rpm. Thereafter, PBS was used to wash the Pep@MNPs-CTC complex under a magnetic field at least thrice to isolate the CTCs. After fixation with ethanol and 5% bovine serum albumin blocking buffer, CTCs were incubated with CD45, CK, and CXCR4 antibodies. Simultaneously, DAPI was used to stain the nucleus. The researchers identified the CK+/CD45- and DAPI-stained CTCs using an Olympus IX73 fluorescence microscope (**Figure S2**). In total, 200 healthy cells from the Capan-1 cell line were resuspended in 2 mL of PBS. Subsequent adsorption, washing, fixation, staining, and identification were performed using the same procedure as that used for blood samples (**Figure S2**).

### Statistical analysis

All data were statistically analyzed using SPSS 23.0 (IBM Corp., Armonk, NY, USA) and Prism 9.0 (GraphPad, La Jolla, CA, USA) software. Continuous variables are expressed as mean (minimum - maximum), and categorical variables are expressed as numbers (percentages). Intergroup comparisons of categorical variables were performed using the nonparametric *Mann-Whitney U* test. The area under the receiver operating characteristic curve (AUROC) was used to evaluate diagnostic efficacy. Kaplan–Meier curves were plotted for survival analysis, and *log-rank* tests were performed. Statistical significance was set at *p* < 0.05 (* *p*<0.05, ** *p*<0.01, *** *p*<0.001, **** *p*<0.0001).

## Results

This section may be divided by subheadings. It should provide a concise and precise description of the experimental results, their interpretation, as well as the experimental conclusions that can be drawn.

### The over-expression of CXCR4 could promote the migration of pancreatic cancer cells *in vitro* and *in vivo*

**Figure 1:**
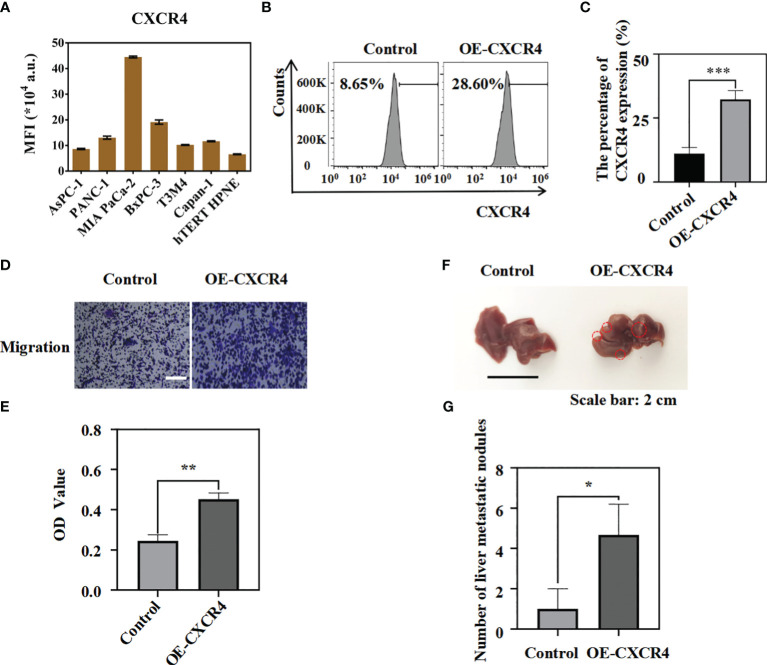
The over-expression of CXCR4 could promote the migration of pancreatic cancer cells *in vitro* and *in vivo*. **(A)** CXCR4 expression in hTERT-HPNE and pancreatic cancer cell lines were detected by flow cytometry analysis. **(B, C)** The protein levels of CXCR4 in AsPC-1 cells transfected with over-expressing CXCR4(OE-CXCR4) were detected by flow cytometry analysis.(**D, E)** Transwell migration assays and quantification of migrated cells of AsPC-1 cells and OE-CXCR4 were performed to evaluate the migrated ability (Scale bar:100 μm). **(F)** The representative photographs of the metastatic tumors in liver from OE-CXCR4/AsPC-1 cells and the control groups were recorded. **(G)** The number of metastatic nodules were measured and compared (n = 3) (unpaired *t* test). *: p<0.05, **: p<0.01, ***: p<0.001.

Previous studies have reported that CXCR4 gene can promote the progression of pancreatic cancer (32, 38, 39). In order to further clarify the role of CXCR4 in pancreatic cancer, *in vitro* and *in vivo* experiments were performed to clarify the effect of CXCR4 on the metastatic ability of pancreatic cancer cells. We found that CXCR4 expression levels were higher in pancreatic cancer cell lines AsPC-1, PANC-1, MIA PaCa-2, BxPC-3, T3M4, and Capan-1 (**Figure 1A**, **Figure S3**). To further clarify the function of CXCR4 in pancreatic cancer, we established the OE-CXCR4/AsPC-1 cell line to stably over-express CXCR4 in AsPC-1 cells (**Figures 1B, C**). Migration experiments suggested that CXCR4 over-expression promoted the migration of AsPC-1 cells (**Figures 1D, E**). To verify the role of CXCR4 in promoting pancreatic cancer cell metastasis *in vivo*, OE-CXCR4/AsPC-1 cells and control AsPC-1 cells were injected into the splenic subcapsular of female nude mice. The number of metastatic nodules of pancreatic cancer liver metastases derived from OE-XCR4/AsPC-1 cells was significantly higher than the control group (**Figures 1F, G**). Therefore, the experimental results suggest that CXCR4 can promote the metastasis of pancreatic cancer *in vivo* and *in vitro*.

### Patients’ characteristics

**Figure 2:**
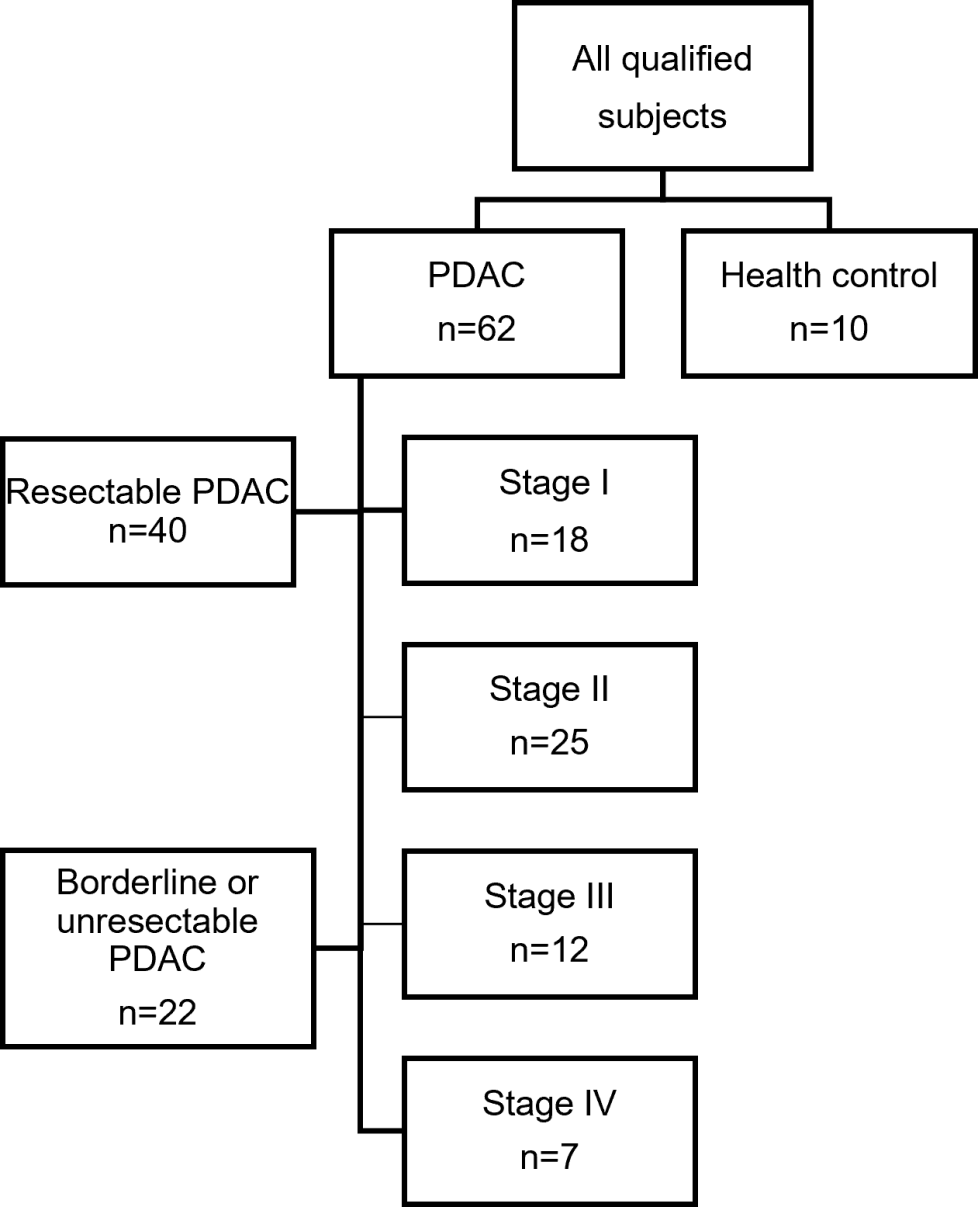
The cohort of the study. (The tumor staging was according to the 8th TNM staging system).

We have validated the function of CXCR4 on promoting the PDAC progression *in vitro* and *in vivo*. To further validate the clinical significance of CXCR4-high expression subsets, we use the peptide-modified nanoparticles(Pep@MNPs platform) to capture and identify the CTC and CXCR4-high CTCs. The median age of the sixty-two patients was 65 years (range, 37–89 years) in our study, and, twenty-two (22/62, 35.5%) patients were men. Forty patients (40/62, 64.5%) underwent radical resection (**Figure 2**), and twenty-seven patients were performed pancreaticoduodenectomy and thirteen patients were performed distal pancreatectomy. The median follow-up time was 17 months in the study, and the median RFS was 16 months in the surgical subgroup. Additionally, twenty-nine (29/40, 72.5%) patients received postoperative gemcitabine-based chemotherapy in the surgical subgroup. Until the final follow-up, 58.1% of all patients were alive, whereas 67.5% of patients in the surgical group were alive. Furthermore, 46.8% (29/62) of all patients had distant metastasis, whereas 40% (16/40) of the patients in the surgical group developed distant metastasis. In the surgical subgroup, ten patients had liver metastases after surgery; three patients suffered from liver metastasis combined with peritoneal metastasis or local recurrence, two patients were diagnosed with lung metastasis; and one patient suffered from a locoregional recurrence.

### The capture and detection of CTCs or HM-CTCs in pancreatic cancer patients

The Pep@MNPs were proved to effectively and precisely capture the CTCs in our precious study (12). In our study, forty-nine patients (49/62, 79.0%) had CTC counts >0/2 mL in the PB, and thirty-one patients (31/40, 77.5%) had CTC counts >0/2 mL in their PVB in the surgical subgroup. Thirty-one patients (31/49, 63.3%) had HM-CTCs in PB, and twenty patients (20/31, 64.5%) had HM-CTCs in PVB. In the surgical subgroup, the CTC counts of PVB (mean value 10/2 mL) were higher than those of PB (mean value 6/2 mL) (*p <*0.05, **Figure S4A**). We observed that the HM-CTC PVB counts (mean value 4/2 mL) were higher than the PB counts (mean value 2/2 mL) (*p* < 0.05, **Figure S4B**), which implied that the CTCs might reflect the real tumor biological information in PVB. The CTC counts differed in tumor location (*p* = 0.054), and they were higher when the tumor was in the body or tail (**Table S1**). We divided the surgical group into high- and low-expression subgroups based on the median number of CTCs and 1/2 mL for HM-CTCs. We also analyzed the clinicopathological differences between both subgroups (**Tables S2**, **S3**). Despite not being statistically significant, we observed tendencies in the CTC PB subgroups in terms of tumor location (*p* = 0.083), perineural invasion (*p* = 0.077), intraoperative bleeding (*p* = 0.083), and HM-CTC PB subgroups in CA19–9 level (*p* = 0.057).

### Diagnostic efficacy and survival analysis in all patients

The diagnostic efficacy of PB CTCs in distinguishing patients with PDAC from healthy participants was compared based on AUROC curves (**Figure S5**). Only CTC-like cells (3/2 mL) were detected in the PB of one healthy participant. The AUROC of PB CTCs for distinguishing PDAC patients from healthy controls was 0.862 (**Figure S5A**). In addition, we tested the diagnostic efficacy of combining PB CTCs with CA19–9 levels, which increased to 0.963 (**Figure S5B**). In the patients’ survival analysis, no significant differences were observed between the high- and low-CTCs subgroups in PB (*p* = 0.13, **Figure S6A**.). In contrast, the prognosis of high HM-CTCs subgroup was worse than that of the low HM-CTCs subgroup in the PVB (*p* = 0.043, **Figure S6B**). The HM-CTCs showed attractive value for diagnostic and prognostic evaluation in pancreatic cancer.

### The prognosis analysis of the CTC or HM-CTC subgroup in the surgical group

**Figure 3:**
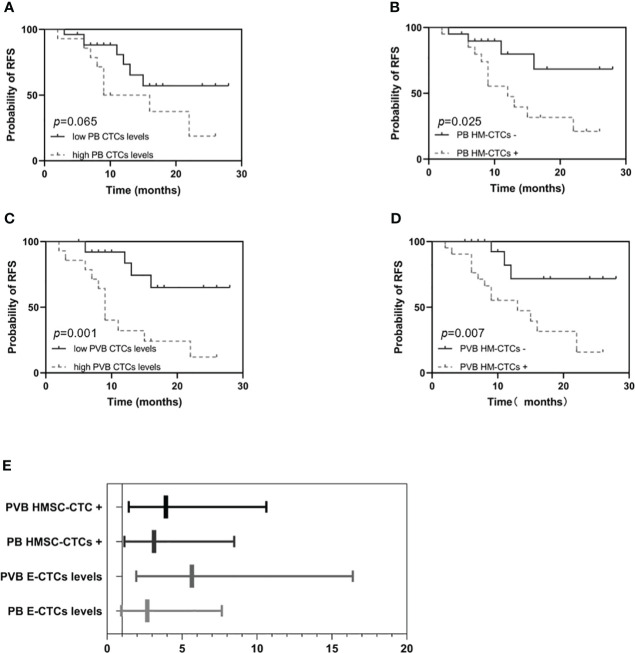
Recurrence free survival and hazard ratios among the surgical group patients (n = 40) by PB or PVB CTC levels and HM-CTCs. RFS in the high or low PB CTCs levels **(A)** and PB HM-CTCs **(B)**. RFS in the high or low PVB CTCs levels **(C)** and PVB HM-CTCs **(D)**.The hazard ratios among the surgical group patients by PB or PVB CTC levels and HM-CTCs **(E)**. *Log-rank* test, *p* value = 0.065 **(A)**, 0.025 **(B)**, 0.001 **(C)**, and 0.007 **(D)**.

**Figure 4:**
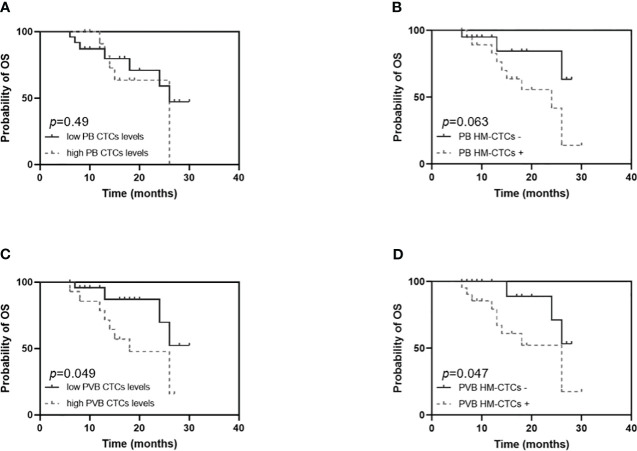
Overall survival **(A-D)** among the surgical group patients (n = 40) by PB or PVB CTCs levels and HM-CTCs. OS in the high or low PB CTCs levels **(A)** and PB HM-CTCs **(B)**. OS in the high or low PVB CTCs levels **(C)** and PVB HM-CTCs **(D)**. *Log-rank* test, *p* value = 0. 49 **(A)**, 0.063 **(B)**, 0.049 **(C)**, 0.047 **(D)**.

We compared RFS and OS based on CTCs and HM-CTCs levels in PB and PVB (**Figures 3**, **4**). Although it was not statistically significant, we observed that the RFS of low CTC subgroup was longer in PB (HR, 2.683; 95% CI, 0.9407–7.652, *p* = 0.065) (**Figure 3A**). The RFS of the low HM-CTC subgroup was longer than that of the high HM-CTC subgroup in PB (HR, 3.126; 95% CI, 1.153–8.475, *p* = 0.025) (**Figure 3B**). In addition, the RFS of the low CTC or HM-CTC sub-group was longer than that of the high-CTC or HM-CTC subgroup in the PVB of surgical group (HR, 5.653, 95% CI, 1.949–16.40, *p* = 0.001; HR, 3.917, 95% CI, 1.444–10.63, *p* = 0.007) (**Figures 3C–E**). The OS analysis between the PB CTC and HM-CTC subgroups was not significant (**Figures 4A, B**). The OS analysis in the low CTC and HM-CTC subgroups was significantly longer than that in the high subgroup in PVB (HR, 3.18; 95% CI, 1.004–10.07, *p* = 0.049; HR, 3.189; 95% CI, 1.013–10.04, *p* = 0.047) (**Figures 4C, D**). Therefore, our results implied that CTCs and HM-CTCs in PVB could effectively predict the RFS and OS.

### HM-CTC accuracy in predicting early recurrence and PDAC liver metastasis

**Figure 5:**
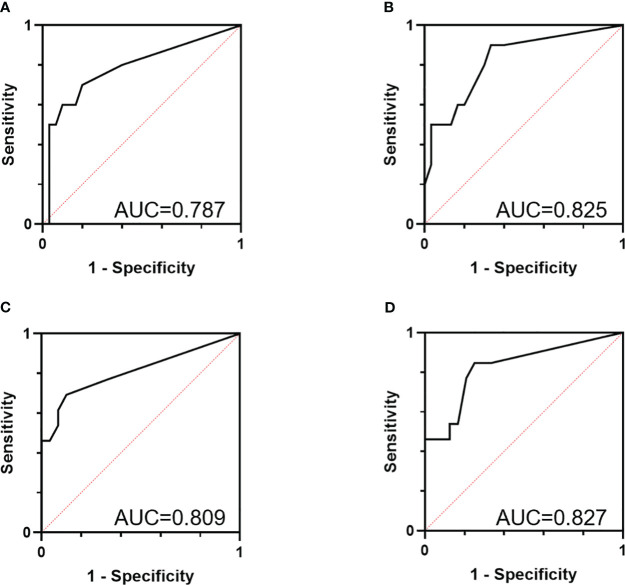
AUC curves to predict the early recurrence **(A, B)** and liver metastasis **(C, D)** of the surgical group based on the HMSC-CTCs in PB and PVB. AUC curves to predict the early recurrence based on the HMSC-CTCs in the PB **(A)** and PVB **(B)**. AUC curves to predict the liver metastasis based on the HMSC-CTCs in the PB **(C)** and PVB **(D)**. HM-CTCs have been depicted (AUC=0.787, 0.825, 0.809, 0.827, n = 40).

We identify the CXCR4-positive CTCs and define them as HM-CTCs. Then we evaluate their accuracy in predicting the early recurrence rate of pancreatic cancer (< 9 months) and liver metastasis (**Figure 5**). We observed that the AUROC of PB HM-CTCs to distinguish early recurrence was 0.787, and the accuracy significantly increased when we tested the AUROC of PVB HM-CTCs (AUROC = 0.825). The AUROC of PB HM-CTC to diagnose liver metastasis was 0.809, and the accuracy increased to 0.827 when we assessed the AUROC of HM-CTCs in PVB. Therefore, the above results implied that preoperative HM-CTCs in the PB and PVB could help evaluate early recurrence and liver metastasis in patients with PDAC, which showed excellent ability to identify patient subpopulation who were prone to suffering early recurrence and metastasis. And the sample size needs to be expanded in the future work to build the prediction model based on the HMSC-CTCs and clarify the accuracy through internal verification and external verification, thus accurately predicting the occurrence of recurrence, liver metastasis and the prognosis of pancreatic cancer patients.

## Discussion

The late diagnosis, early recurrence and metastasis contributed to the poor prognosis of pancreatic cancer. Tumor biological behavior varies in different pancreatic cancer patients. How to divide pancreatic cancer patients into different subgroups according to the biological behavior of tumors and take different treatment measures is an urgent clinical problem to be solved. The current detection technique could not precisely diagnose and evaluate the prognosis. But the recurrence and early occult metastasis occurred when the “CTC” shed from the tumor, which act as important biomarkers (10, 40). Therefore, CTCs are important and classical liquid biomarkers to evaluate metastasis and recurrence in pancreatic cancer patients. We found that the novel Pep@MNP detection system was feasible for pancreatic cancer diagnosis.

CellSearch® detection system was the FDA-approved CTC detection method which could capture the CTCs by targeting the epithelial cell marker “EpCAM” through antigen-antibody specific binding. But the detection rate was poor in pancreatic cancer (12). Peptides have the advantage of small molecular weight and low cost. We have modified the EpCAM-targeted peptide on the surface of magnetic beads to capture more CTCs. Our research group developed the novel Pep@MNP detection system that was verified in lung and liver cancer (13, 14, 41). To further explore its application in pancreatic cancer, we used the Pep@MNP system to detect CTCs in pancreatic cancer and focused on the HM-CTC subpopulation. The positivity rates of CTCs in PB and PVB were 79.0% (49/62) and 77.5% (31/40). The Pep@MNP detection system exhibited the higher CTCs capture efficiency, which proved the excellent targeting ability of the Pep@MNPs assay to the EpCAM on CTCs and to exclude from the interference from blood cells (13). We find that the AUC of CTCs in PB for pancreatic cancer is 0.862, and the AUC increased to 0.963 when combined with CTCs and CA19–9. Therefore, this study confirms that the CTCs captured by the Pep@MNP detection system was feasible for diagnosing pancreatic cancer.

Pancreatic cancer sheds tumor cells into the portal vein, which might be filtered and colonized by the liver, and specific subtypes of tumor cells might be prone to colonize in the liver and form the tiny metastatic foci. Therefore, portal vein blood (PVB) CTCs in pancreatic cancer can accurately and intuitively reflect biological characteristics, and their diagnostic and prognostic values are significantly better than those of PB (16). The pancreatic veins flow through the portal vein of the liver, and the CTCs could fall into the portal vein. The CTCs subtypes could be filtered and colonized the liver, thus promoting liver metastasis. The PVB CTC count is higher than the PB-CTC count and can strongly reflect the real biological behavior (42–44), which was consistent with our results. Our study also observed that PVB CTCs and HM-CTCs counts were higher than the PB CTCs counts (*p <*0.05, *p <*0.05). Bissolati et al. (44) revealed that the incidence of liver metastasis in patients with CTCs was significantly higher than that in those without CTCs (53% vs. 8%, *p* = 0.038). Liu et al. (24) reported that the CTC counts in the portal vein were highly associated with intrahepatic metastases. However, no significant difference was observed in postoperative survival between the high- (>8/2 mL) and low-CTC (≤8/2 mL) subgroups (*p* = 0.13) in our research. The recurrence and metastasis rates of the higher portal CTC count (>10/2 mL) were significantly higher than those of the lower portal CTC count (≤10/2 mL) (*p* = 0.001), with shorter postoperative survival (*p* = 0.049). Therefore, it is feasible to apply the Pep@MNP detection system to capture CTCs for the prognostic evaluation in pancreatic cancer patients, but PVB CTCs are more precise in predicting postoperative metastasis and survival of pancreatic cancer.

Our previous study revealed that CTC phenotype analysis based on EMT or hENT-1 helped establish new prognostic biomarkers in patients with resectable cancer receiving gemcitabine-based adjuvant chemotherapy (45). In this study, we focus on the predictive efficacy of CTCs and CTCs subsets in distant metastasis. We focused on the CXCR4 gene which was over-expressed in pancreatic cancer. We further verified the function of CXCR4 in promoting the liver metastasis of pancreatic cancer, which was consistent with previous research. So, we defined the CXCR4 over-expressed CTC subpopulation as HM-CTCs. Studies have revealed that patients with high CXCR4 expression have a shorter OS and a higher risk of liver metastasis than those with low expression (46). It is consistent with the results reported in our study. When CTCs fall from pancreatic cancers into the liver, HM-CTCs (CXCR4+CTC) may directly colonize the liver to metastasize. Our study observed that the number of HM-CTCs in PVB were more than HM-CTCs in PB, suggesting that some HM-CTCs might colonize in the liver. In the surgical group, the RFS and OS of the low-CTC or HM-CTC subgroups was longer than that of the high-CTC or HM-CTC subgroups in PVB. So, we defined the CXCR4 over-expressed CTC subpopulation as HM-CTCs, which could identify PDAC patients with poor prognosis.

Pancreatic cancer was easy to relapse and metastasize, and early recurrence is a key factor in determining patient prognosis. Therefore, it is necessary to monitor patients who easily relapse preoperatively according to the biological characteristics of the tumor to guide clinical treatment. CTCs were reported to be associated with prognosis and the disease stage (47). Javed et al. (19) reported that a positive CTCs were a significant risk factor for late recurrence in multivariate analysis (*p* = 0.024). Park et al. reported that positive CTC was an independent risk factor for early (*p* = 0.027) and systemic recurrence (*p* = 0.033) (48). Zhang et al. reported that preoperative CTC-positive was independent risk factor for disease-free survival (*p* < 0.05) and for OS in pancreatic cancer patients (*p* < 0.05) (49). Hugenschmidt et al. found that the presence of preoperative CTCs was associated with earlier metastasis and shorter OS (50), but the CTCs were detected in only about 7% of patients based on FDA-approved CellSearch® test. Bissolati et al. reported that PVB CTCs were detected in 45% of the patients and the liver metastasis rate of PVB CTC-positive pancreatic cancer patients was higher than CTC-negative patients (*p* = 0.038) at 3-year follow-up (44). But no correlation was found between CTCs and OS. In our study, the positivity rates of CTCs in PB and PVB were 79.0% (49/62) and 77.5% (31/40) based on the Pep@MNP detection platform, which were associated with the recurrence, liver metastasis and OS.

CTCs contain abundant biological information, and identifying CTC subsets with high malignancy based on specific markers are more able to select patients with poor prognosis. Cheng et al. reported that folate receptor-positive CTCs were independent risk factor for distant metastasis (*p* = 0.014) and early recurrence (*p* = 0.013), but they could not predict OS in the non-surgical group (*p* = 0.220) (51). The detection of vimentin-positive and cytokeratin-positive transitional CTCs was predictive of recurrence (*p* = 0.01) and shorted RFS (10). And in patients who did not experience effective adjuvant therapy, postoperative positive trCTCs was associated with poorer RFS (20). Rizzo et al. reported that the bone metastasis rate of CXCR4-positive CTCs in neuroendocrine tumor patients was 56% compared to 35% in patients without bone metastasis (*p* = 0.18),which may be involved in CTC osteotropism and predicting the bone metastasis (52). We analyses that CXCR4-positive CTCs may be attracted to the liver and lung,which produce large amounts of the chemokine CXCL12. We verified the pro-oncogenic role of CXCR4 *in vitro* and *in vivo*, and define the CXCR4-positive CTCs as the HM-CTCs. Then we evaluated the accuracy to predict early recurrence rate for pancreatic cancer based on the HM-CTCs counts. Our team previously reported a multicenter retrospective study in which 9 months was the best threshold to distinguish early from late recurrence (53). We observed that the AUC of HM-CTCs in PB for distinguishing early recurrence was 0.787, and the accuracy significantly increased when we tested the AUC of PVB HM-CTCs (0.825). Therefore, the preoperative detection of HM-CTCs in PB or PVB might help identify high-risk pancreatic cancer subpopulation who were prone to suffering early recurrence and metastasis. But we could not ignore the influence of neoadjuvant therapy on CTCs counts of patients with pancreatic cancer, which is unclear. Gemenetzis et al. (32) reported that the median CTCs in patients who had undergone neoadjuvant therapy were significantly lower than chemo-naïve PDAC patients before surgery in fifty-seven patients undergoing surgery. But Poruk et al. (33) reported that there existed no difference in the sixteen PDAC patients who received neoadjuvant therapy. Therefore, the stable or decreased CTC counts might imply the effective response to neoadjuvant therapy (34), and it is necessary to further explore whether neoadjuvant chemotherapy will affect the accuracy of predictions of preoperative HMSC-CTCs on early recurrence.

Existing metastatic cancer spread refers to the sequence of events following a successful primary tumor seeding event, followed by a metastasis cascade (54). In this case, metastasis prevention therapy combined with tumor-killing therapy may benefit patients by avoiding further cancer spread and reducing tumor burden (54). Our study observed a high incidence of recurrence and distant metastasis in pancreatic cancer patients with high HM-CTCs. CXCR4-targeted tumor therapy has promising applications in pancreatic cancer. Bockorny et al. (34) reported promising results wherein the combined blockade of CXCR4 and PD-1 enhanced the chemotherapeutic effect in metastatic PDAC (NCT02826486). Therefore, the HM-CTCs counts could help identify PDAC patients who might benefit from the combination of CXCR4-targeted therapy and immunochemotherapy. It is necessary to early identify the HM-CTCs high subgroup to guide the clinical treatment, thus improving the clinical precise diagnosis and treatment of pancreatic cancer.

This study has a few limitations. Firstly, further studies with larger sample sizes are needed to verify the clinical value of this study since this investigation is still based on the small sample size and limited follow-up. In our study, most of patients were at either stage 1 or 2 disease and follow-up period should be prolonged to one year and more to evaluate the predictivity of CTCs and HMSC-CTCs on the recurrence and liver metastasis. And our results could not effectively represent the clinical significance of the patients at advanced stages. More patients at advanced stages should be enrolled in the subsequent study. Secondarily, benign pancreatic disease was not included as a control when evaluating the diagnostic value of CTCs for pancreatic cancer. Pancreatic cancer is difficult to differentiate from chronic pancreatitis, intraductal papillary mucinous neoplasms and autoimmune pancreatitis, which should be enrolled in the subsequent study. Finally, we did not monitor dynamic changes of HM-CTCs counts when assessing the correlation between HM-CTCs and distant metastasis in pancreatic cancer. Peripheral blood should be sampled at different time points before and after surgery to evaluate the dynamic changes of HMSC-CTCs over time and treatment. The effect of adjuvant therapy and neoadjuvant therapy (duration, type, and dosage) on CTC should also be considered in subsequent studies.

In conclusion, our study observed that CXCR4 could promote the migration of pancreatic cancer *in vitro* and *in vivo*. And, it was feasible to apply the Pep@MNPs assay system to detect CTCs in the PB and PVB of pancreatic cancer patients. The CTCs can be used as diagnostic biomarkers, and their combination with CA19–9 can significantly increase diagnostic accuracy. Moreover, we defined the CXCR4 over-expressed CTCs subpopulations as HM-CTCs, which could identify high-risk pancreatic cancer subpopulation who were prone to suffering early recurrence and metastasis. And they could be used as potential biomarkers to screen patients who can benefit from the combination of CXCR4-targeted therapy and immunochemotherapy. Therefore, the Pep@MNPs detection system showed well application prospects for guiding the diagnosis and prognosis evaluation of pancreatic cancer patients.

## Data availability statement

The raw data supporting the conclusions of this article will be made available by the authors, without undue reservation.

## Ethics statement

The studies involving humans were approved by Peking University First Hospital ethics committee. The studies were conducted in accordance with the local legislation and institutional requirements. The participants provided their written informed consent to participate in this study.

## Author contributions

XC: Data curation, Formal analysis, Investigation, Methodology, Writing – original draft. XZ: Data curation, Formal analysis, Methodology, Writing – original draft. SZ: Data curation, Formal analysis, Methodology, Writing – review & editing. MW: Methodology, Writing – review & editing. PL: Methodology, Writing – review & editing. YM: Data curation, Methodology, Writing – review & editing. CW: Methodology, Writing – review & editing. XT: Conceptualization, Methodology, Supervision, Writing – review & editing. YLY: Conceptualization, Funding acquisition, Methodology, Resources, Supervision, Writing – review & editing. YMY: Funding acquisition, Resources, Writing – review & editing.
