# Supplementary material for: Stem cell-like circulating tumor cells identified by Pep@MNP and their clinical significance in pancreatic cancer metastasis
Source: Front Oncol. 2024 Jun 25;14:1327280. doi: 10.3389/fonc.2024.1327280 (PMC11231205; doi:10.3389/fonc.2024.1327280)
Supplement: Supplementary file 1 [file DataSheet_1.docx]

**
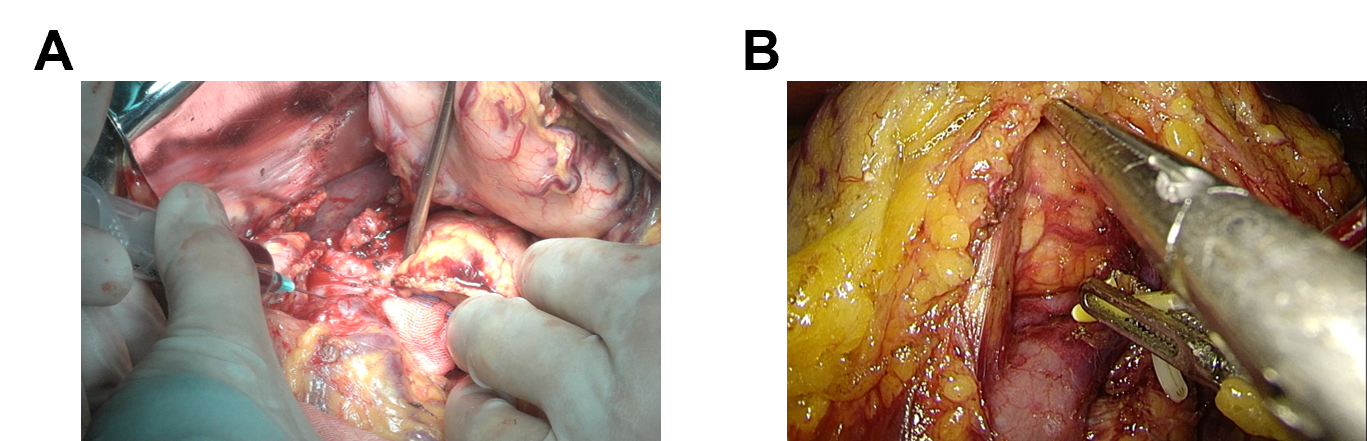
**

**Figure S1** PVB was drawn in open (A) and laparoscopic (B) distal pancreatectomies.

**
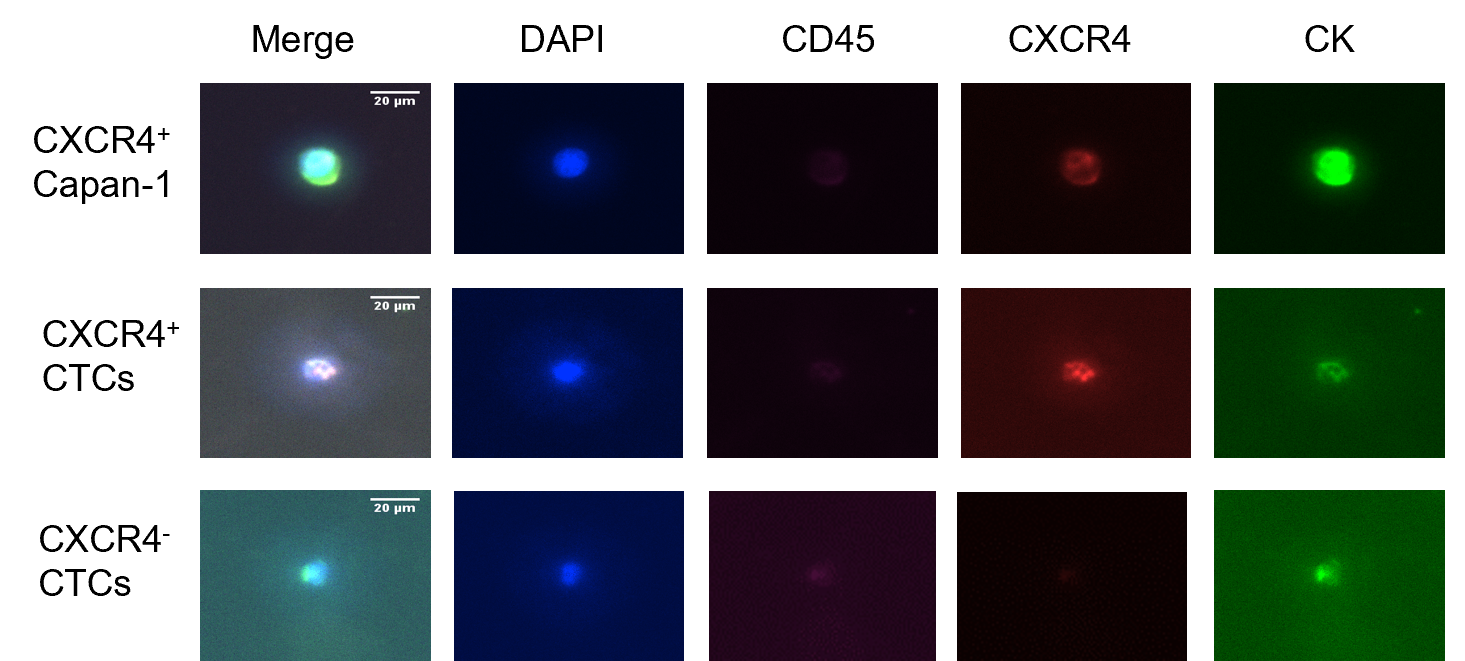
**

**Figure S2** The Capan-1 cells and CXCR4+/CXCR4- CTCs were captured by Pep@MNPs.


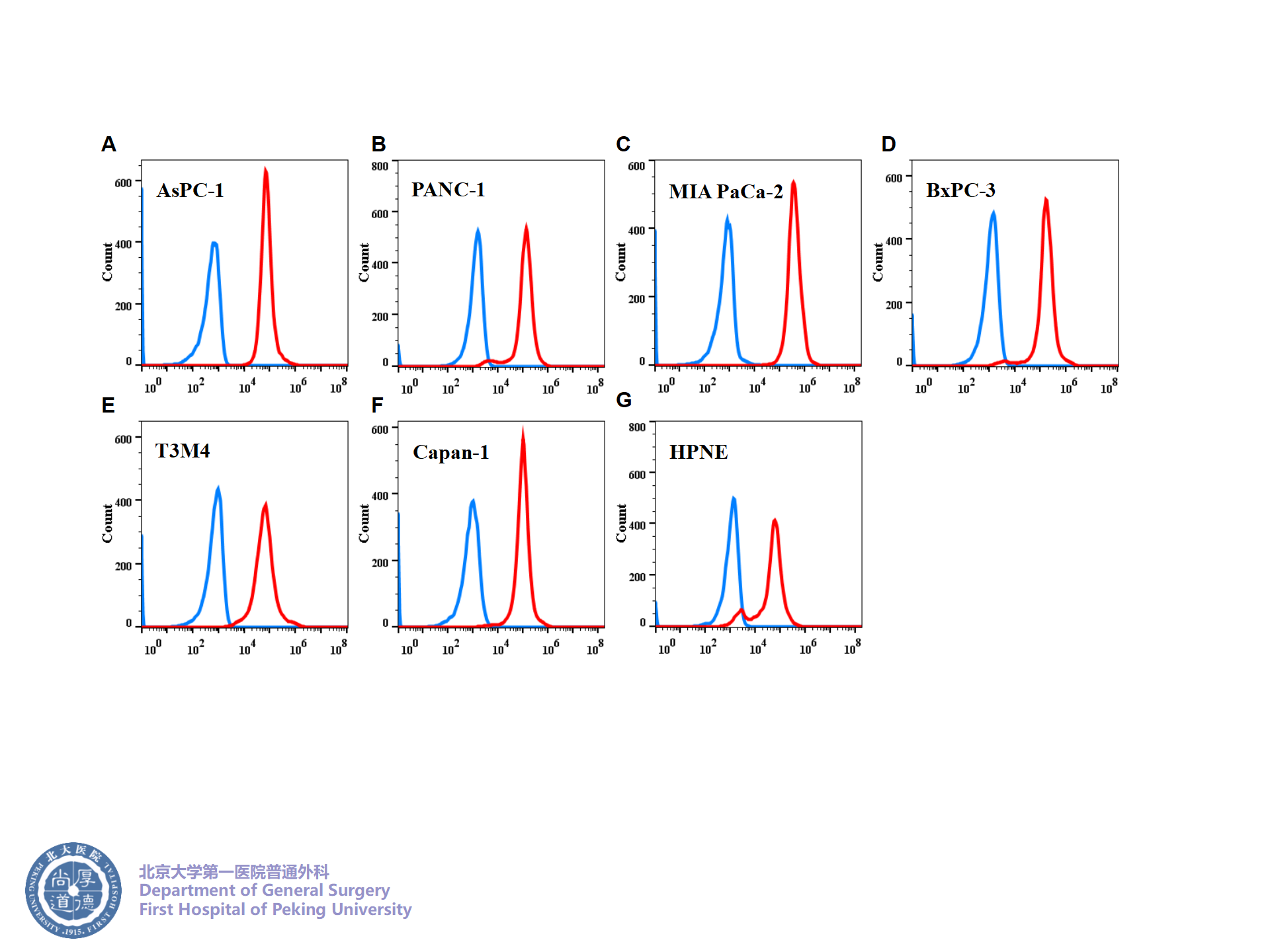


**Figure S3** The expression of CXCR4 was evaluated in pancreatic cancer cell lines AsPC-1 (A), PANC-1 (B), MIA PaCa-2 (C), BxPC-3 (D), T3M4 (E), and Capan-1 (F) and normal HPNE cells (G).

**
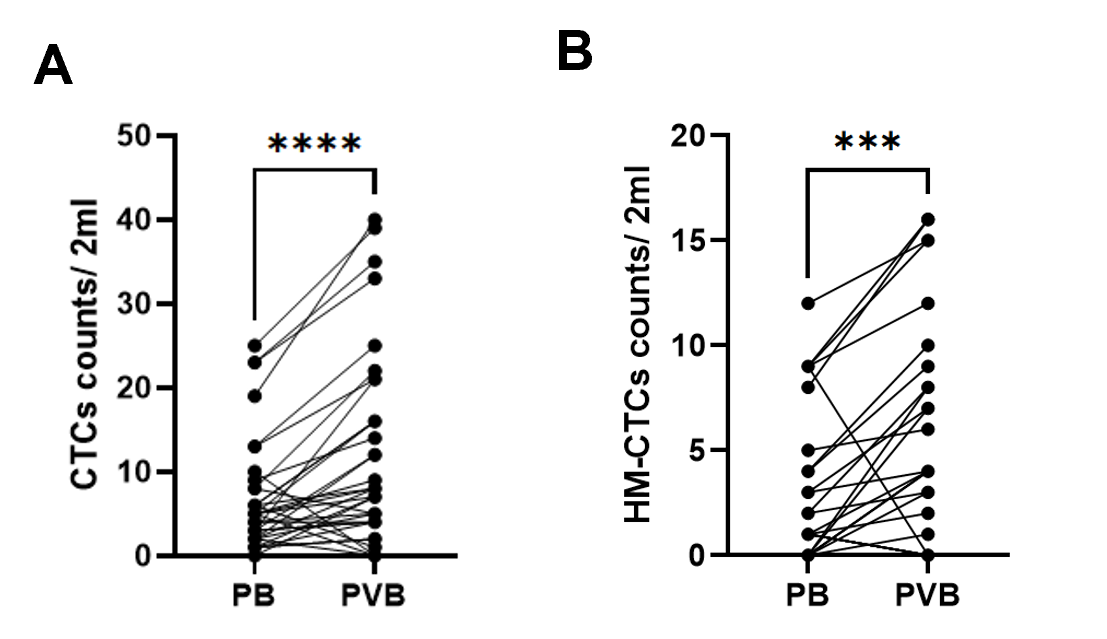
**

**Figure S4** CTCs counts (A) and HM-CTCs counts (B) showed significant differences between PB and PVB in the surgical group (*Wilcoxon matched-pairs signed rank* test, n = 40).

**
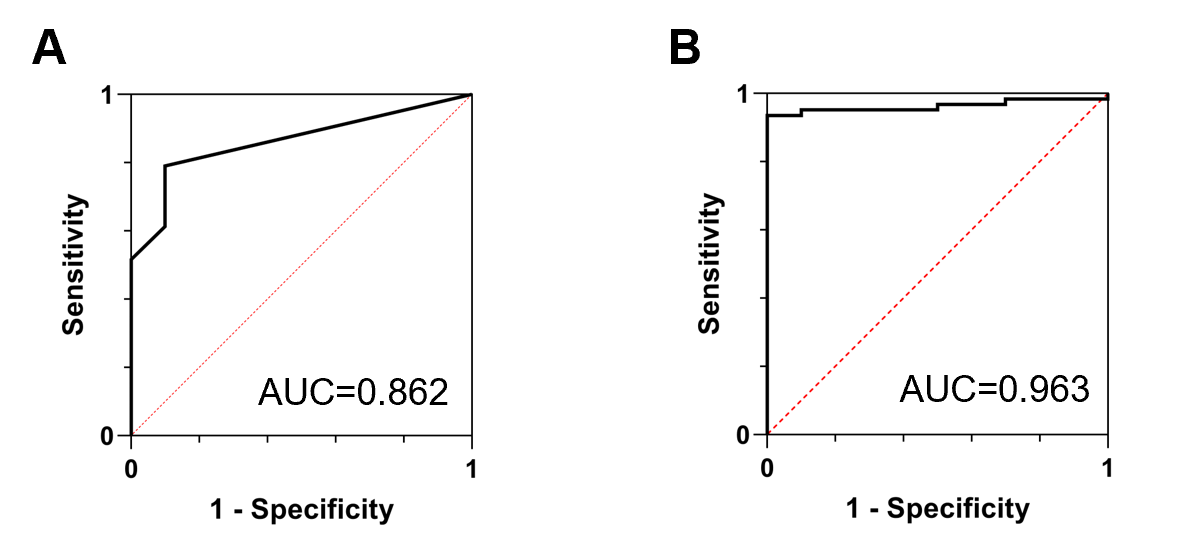
**

**Figure S5**. ROC curves to diagnose pancreatic cancer based on PB CTCs (A) counts and PB CTCs (B) counts combined with CA19-9 have been depicted (AUC=0.862, 0.963; n = 62).

**
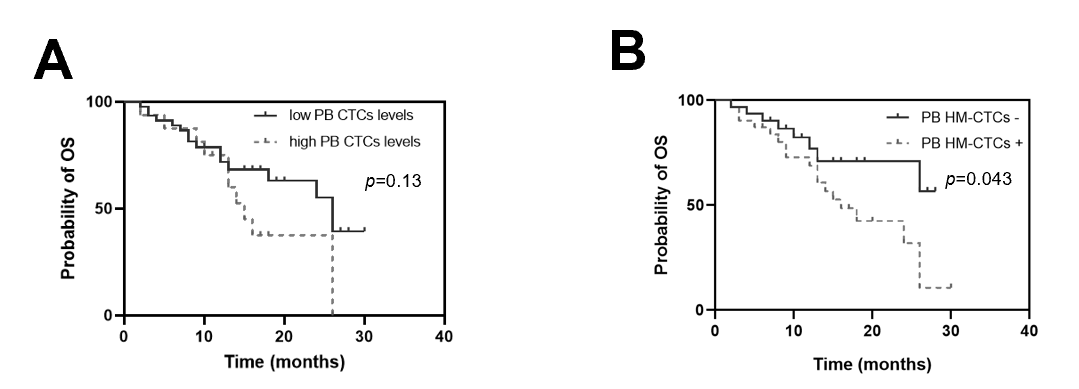
**

**Figure S6**. Overall survival among all patients (n = 62) by PB CTCs and HM-CTCs levels. *Log-rank* test, *p* value = 0. 13 (A), 0.043 (B).

**Table S1** Demographics, clinicopathological characteristics and PB CTCs counts of all patients

| Characteristics | All patients, n = 62 | CTCs counts/2mL | *p* value |
| --- | --- | --- | --- |
| Sex |  |  | 0.407 |
| Male | 22 | 4.5 (0.75, 19.75) |  |
| Female | 40 | 3 (1, 8) |  |
| Age  ≥65 | 32 | 3.5 (0.25, 7.5) | 0.505 |
| <65 | 30 | 5 (1, 10.75) |  |
| CA19-9 level, U/mL  ≥200  <200 | 34  28 | 4.5 (0.75, 9.25)  3 (1, 9) | 0.749 |
| Tumor location |  |  | 0.054 |
| Head  Body or tail  AJCC stage  Ⅰ  Ⅱ  Ⅲ  Ⅳ | 42  20  18  25  12  7 | 3 (0, 6.5)  6 (2.5, 10)  2.5 (1, 6.5)  4 (2.5, 13)  1 (0.25, 12)  6 (0, 22) | 0.562 |

**Table S2** Correlations between CTC levels and clinicopathological characteristics in the surgical group

| Characteristics | High PB CTCs levels | Low PB CTCs levels | *p* value | High PVB CTCs levels | Low PVB CTCs levels | *p* value |
| --- | --- | --- | --- | --- | --- | --- |
| Sex  Male | 6 | 6 | 0.193 | 5 | 7 | 0.563 |
| Female | 8 | 20 |  | 9 | 19 |  |
| Age  ≥65 | 8 | 17 | 0.169 | 7 | 16 | 0.481 |
| <65 | 6 | 9 |  | 7 | 10 |  |
| CA19-9 level, U/mL  ≥200  <200 | 8  6 | 14  12 | 0.842 | 8  6 | 14  12 | 0.842 |
| Tumor location |  |  | 0.083 |  |  | 0.750 |
| Head  Body or tail | 7  7 | 6  20 |  | 9  9 | 18  8 |  |
| Tumor grade  Poor  Moderate and well | 6  8 | 12  14 | 0.842 | 6  8 | 12  14 | 0.842 |
| Lymphatic status  Positive  Negative  Perineural invasion  Positive  Negative  Vessel invasion  Positive  Negative | 9  5  11  3  8  6 | 13  13  25  1  10  16 | 0.386  0.077  0.257 | 10  4  12  2  8  6 | 12  14  24  2  10  16 | 0.125  0.507  0.257 |
| Intraoperative bleeding  High  Low | 7  7 | 6  20 | 0.083 | 6  8 | 7  19 | 0.305 |

**Table S3** Correlations between HM-CTCs and clinicopathological characteristics in the surgical group

| Characteristics | PB HM-CTCs + | PB HM-CTCs - | *p* value | PVB HM-CTCs + | PVB HM-CTCs - | *p* value |
| --- | --- | --- | --- | --- | --- | --- |
| Sex |  |  | 0.168 |  |  | 0.629 |
| Male | 8 | 4 |  | 7 | 5 |  |
| Female | 12 | 16 |  | 14 | 14 |  |
| Age |  |  | 0.110 |  |  | 0.184 |
| ≥65 | 9 | 14 |  | 10 | 13 |  |
| <65 | 11 | 6 |  | 11 | 6 |  |
| CA19-9 level, U/ml |  |  | 0.057 |  |  | 0.775 |
| ≥200 | 14 | 8 |  | 12 | 10 |  |
| <200 | 6 | 12 |  | 9 | 9 |  |
| Tumor location |  |  | 0.311 |  |  | 0.906 |
| Head | 12 | 15 |  | 14 | 13 |  |
| Body or tail | 8 | 5 |  | 7 | 6 |  |
| Tumor grade |  |  | 0.204 |  |  | 0.119 |
| Poor | 7 | 11 |  | 7 | 11 |  |
| Moderate and well | 13 | 9 |  | 14 | 8 |  |
| Lymphatic status |  |  | 0.527 |  |  | 0.356 |
| Positive | 11 | 9 |  | 13 | 9 |  |
| Negative | 9 | 11 |  | 8 | 10 |  |
| Perineural invasion |  |  | 1.000 |  |  | 0.342 |
| Positive | 18 | 18 |  | 18 | 18 |  |
| Negative | 2 | 2 |  | 3 | 1 |  |
| Vessel invasion |  |  | 0.525 |  |  | 0.324 |
| Positive | 10 | 8 |  | 11 | 7 |  |
| Negative | 10 | 12 |  | 10 | 12 |  |
| Intraoperative bleeding |  |  | 0.311 |  |  | 0.141 |
| High | 8 | 5 |  | 9 | 4 |  |
| Low | 12 | 15 |  | 12 | 15 |  |
